# Factors influencing the mental health of autistic children and teenagers: Parents’ observations and experiences

**DOI:** 10.1177/13623613231158959

**Published:** 2023-03-15

**Authors:** Suzanne Mukherjee, Bryony Beresford

**Affiliations:** University of York, UK

**Keywords:** adolescence, autism, children, mental health, parents, qualitative research, teenagers

## Abstract

The high prevalence of mental health and behaviour problems among autistic children and adults is an issue of concern to the autism community. Many studies have been undertaken to identify the factors that protect against/or increase the risk of such difficulties. However, this research is dominated by quantitative observational studies. In this study, we sought to investigate the same issue using a qualitative research approach, positioning parents as experts and eliciting their theories as to what influenced their child’s mental health from diagnosis to the late teenage years. In-depth interviews were undertaken with 33 parents (30 mothers, 3 fathers) of 31 autistic teenagers (21 males, 10 females) aged between 15 and 19 years (median 17 years) purposively sampled from an existing cohort (QUEST). Parents believed a wide range of child-centred, developmental and socio-environmental factors had played a role in their child’s mental health, alongside life events involving loss and separation. A number of these factors have received little or no research attention to date (e.g. aspects of the school environment). The findings have important implications, highlighting factors that should be given priority in future research, as well as interventions needed to support the mental health of autistic teenagers.

**Lay abstract:**

Autistic people are more likely to experience mental health difficulties compared to neurotypical people. It is very important that we understand what increases the risk for mental health difficulties, and what helps to protect against them. So far, research on this for children and young people has only investigated a small number of factors and these have been chosen by researchers and clinicians. This study took a different approach in which parents’ expertise in their children was recognised. Parents were asked to tell the story of their autistic teenagers’ mental health from diagnosis in early childhood through to the present, and to explain the ‘theories’ they developed about what affected their child’s mental health – positively and negatively – and how. Parents believed a wide range of factors played a role. These include: (1) aspects of their child (e.g. their autistic traits, intelligence); (2) aspects of their surroundings (e.g. the efforts parents make to prevent and respond to their child’s difficulties, features of the school they attend, availability of social activities); (3) changes their child experienced growing up (e.g. puberty, awareness of being autistic); and (4) life events involving loss and separation. Many of the factors parents identified as important have received little or no research attention to date. The findings suggest issues that should be considered in future research and reveal ways that support for parents and autistic children and teenagers can be improved.

## Background

Mental health is a top research priority for the autism community (Autistica, 2015; Roche et al., 2020). This is not surprising given the evidence of a significantly higher prevalence of mental health and behavioural problems (MHBPs) among autistic children, young people and adults compared to the general population (Lai et al., 2019; Roche et al., 2020; Tsai, 2014; Wigham et al., 2017). The mental health of teenagers is a particular concern, with longitudinal studies reporting that, while behavioural problems and attention-deficit hyperactivity disorder (ADHD) present in childhood decline, anxiety remains high or increases, and there is a rise in depression (Gotham et al., 2015; McCauley et al., 2020; Rai et al., 2018).

Being autistic does not, however, mean that individuals necessarily experience MHBPs (Gotham et al., 2020) and numerous quantitative studies have sought to identify factors that protect against and/or increase the risk of MHBPs among autistic children and young people. These indicate that, as well as severity of autism, other factors are at play. These include cognitive factors known to be associated with autism (e.g. verbal IQ; theory of mind capacity; perceptual processing skills; alexithymia), other child-centred factors (e.g. adaptive functioning skills) and socio-environmental factors (Carter Leno et al., 2018; Chandler et al., 2016; Colvert et al., 2021; Hollocks et al., 2014; Lundström et al., 2011; McCauley et al., 2020; Milosavljevic et al., 2016). Research on socio-environmental factors has focused primarily on the home, and particularly parent-centred factors, finding that MHBPs are associated with parental psychological distress, parenting style and ‘household chaos’ (Baker et al., 2011; Breider et al., 2022; Maljaars et al., 2014; McRae et al., 2018; Midouhas et al., 2013; Simonoff et al., 2013; Woodman et al., 2015, 2016; Yorke et al., 2018). Further factors identified include size of peer networks, experiences of bullying, socio-demographic factors (e.g. household income; family size) (Hollocks et al., 2021) and exposure to adverse life events (Taylor & Gotham, 2016).

While quantitative observational studies have progressed understanding about factors increasing the risk of developing MHBPs (or conversely protecting against MHBPs), there are also benefits to examining this question from a qualitative paradigm (Agius, 2013; Bölte, 2014; Williams et al., 2019) and positioning parents as experts (Kruithof et al., 2020). Here, real-world experiences can be used to develop theory using an inductive, rather than a deductive, approach. This may potentially serve to identify factors neglected in past research, hidden from or unobserved by clinic or school-based autism professionals. To date, few studies have sought to do this and, where undertaken, the research has been specific in focus. For example, the role of only autism spectrum disorder (ASD) symptoms (Ozsivadjian et al., 2012), the child’s anxiety management strategies (Clark & Adams, 2020) or parental support practices (Adams et al., 2018). This article reports a qualitative study which sought to elicit parents’ ‘theories’ of their autistic child’s mental health from the point of diagnosis to the late teenage years, including the factors which they believed influenced the development, or not, of MHBPs during this period. The study is part of a programme of research on improving mental health outcomes of autistic people (see https://iamhealthkcl.net/).

## Methods

### Study aims and theoretical framework

The aim was to elicit parents’ descriptions of, and theories regarding, positive and negative changes in their teenage autistic child’s mental health and behaviour from the point of diagnosis in early/mid-childhood to the time of study participation, and the factors contributing to these changes. Its theoretical framework was the transactional model of development (Sameroff, 2010) which argues that developmental outcomes arise from a bi-directional dynamic interplay between the child’s biological (e.g. genetic/neurodevelopmental make-up) and psychosocial self (e.g. communication skills) and features of the child’s socio-ecological environments (e.g. family, peers, school, neighbourhood), with developmental changes (e.g. physical, cognitive) moderating the presence or influence of these factors on outcomes. This aligns with a growing recognition of the need for autism research, and interventions, to engage with the complexity of factors and processes which may affect the development and well-being of autistic children and young people (Binns & Oram Cardy, 2019; Mills et al., 2022; Stallworthy & Masten, 2022).

### Study design

This was a cross-sectional ‘generic qualitative’ study (Bradbury-Jones et al., 2017; Patton, 1990), informed by phenomenological and narrative approaches to qualitative research.

### Sampling

The target sample was parents of 30 older autistic teenagers with different MHBP trajectories since diagnosis. Parents were recruited from an existing UK autism research cohort (QUEST) initially recruited in 2008 following diagnosis (Chandler et al., 2016; Salazar et al., 2015). To date, data have been collected at three time-points: at recruitment (Wave 1: 4–9 years), 7 years later (Wave 2: 11–15 years; *n* = 211) and 9 years later (Wave 3: 13–17 years). At each wave, data were collected on autism severity, MHBPs and IQ.

The QUEST Team provided the research team with an anonymised data set of families who had previously agreed to be contacted about additional associated studies (*n* = 186). A purposive sampling strategy was used to ensure representation of a range of MHBP trajectories. A single indicator of MHBPs was used, with children categorised according to clinical cut-off points (Waves 1 and 2: Developmental Behaviour Checklist (Einfeld & Tonge, 1995); Wave 3: Strengths and Difficulties Questionnaire (https://sdqinfo.org/). The aim was to represent the eight possible MHBP trajectories within the sample (e.g. above clinical cut-off at Waves 1–3; above at Wave 1 and below at Waves 2 and 3, etc.). Within each trajectory we sought equal representation of children with and out learning difficulties (IQ < 70, IQ > 70) and gender (see Supplementary File 1 for sampling frame).

### Recruitment

Recruitment took place around 2 years after Wave 3, between September 2019 and February 2020. Initial contact was a telephone call from the QUEST Team which sought consent to send a study recruitment pack, which included a response form for returning direct to the research team. Receipt of a response form triggered a telephone call to provide further information and, if the parent wished, make arrangements for an interview.

Forty-one of 126 parents returned a response form, of whom 37 were contacted and 31 recruited. In a few instances, a partner additionally took part and a joint interview was conducted. Of the remaining respondents, one declined, two were uncontactable and four agreed to be interviewed but could not be contacted at the arranged time.

### Data collection

Data was collected via an in-depth interview conducted by one researcher (S.M.). Parents were offered the choice of a telephone (*n* = 29, including two joint interviews) or face-to-face interview (*n* = 2, including one joint interview). The interview started with the consent process which was audio-recorded.

The main section of the interview used narrative techniques (i.e. asking parents to ‘tell their story’ of their child’s mental health since diagnosis) because the process of recall, sequencing and narration support meaning-making and the articulation of explanations or reasons for why things have happened (Jovchelovitch, 2000). Thus, the interview comprised the following:

- Background and contextual information collected using semi-structured questions
- The parent’s story of their child’s mental health: here the parent was asked to give an account of their child’s moods, behaviour and mental health from the point of diagnosis and continuing to the present day. Researcher prompts were minimal and used simply to move the story on or seek clarification. For further details, see Supplementary File 2.
- Eliciting and developing explanations: in this phase parent’s explanations or ‘theories’ about their child’s mental health trajectory were elicited, with probes used to explore the potential bio-psychological and socio-ecological factors involved.

Interviews were audio-recorded and transcribed verbatim. The interview duration was between 34 and 192 min (median 74 min). Field notes were made immediately following the interview which, for the telephone interviews, drew on detailed notes made during the interview. These were used to support data immersion and development of the coding framework.

### Data analysis

Data were analysed using a thematic analysis approach (Miles et al., 2019; Spencer et al., 2014). Steps involved: data immersion (including the writing of field notes and a ‘pen portrait’ (Holloway & Jefferson, 2000) of the themes emerging from the interview, which was later checked against the interview transcript); development of an analytical (or coding) framework (including testing of the framework on a sub-set of transcripts); in-line coding of transcripts; extraction of coded data into EXCEL charts (extracts comprised summaries of coded data and verbatim quotes); and scrutiny of data charts to produce analytical notes and graphical data displays. The analytical framework was informed by the study’s theoretical framework and included *a priori* and emergent themes. A key objective of data analysis was to examine and compare data *within* each participants’ accounts in order to track changes over time (in terms of mental health and the factors impacting mental health), and also *between* study participants to identify patterns and differences between sub-groups of study participants (e.g. young people with and without LD, males and females, mainstream vs special school). SM led on all stages of the analytical work, with analytical outputs regularly shared and discussed with BB across the process (see Supplementary Files 3 and 4 for further details on analysis and final coding framework).

### Community involvement

Two advisory panels, one comprising autistic adults without LD (including autistic young adults), and one of parents of autistic children, supported the study and the wider programme of work. For the present study, this included providing feedback on preliminary findings emerging from the research, further data analysis and dissemination.

### The research team

We are female, applied social science researchers, with academic backgrounds in psychology. We are neurotypical and, while both parents, do not have personal experience of having an autistic child. Our academic backgrounds, and commitment to applied research, informed the research objectives and theoretical framework used. During interviews, we strived for a position of ‘empathetic neutrality’ (Ormston et al., 2014) and used our advisory groups, and meetings with the wider research programme team, to challenge assumptions and ensure data analysis was not selective.

### Ethical approval

NHS Research Ethics Committee (reference number: 18/WS/0204) approved the study.

### Data policy statement

Study data are to be archived with the University of York Research Data Service. Access to available anonymised data may be granted following review by the corresponding author.

## Findings

### Study participants

Thirty-three parents (30 mothers, 3 fathers) of 31 autistic teenagers (21 males, 10 females) aged between 15 and 19 years (median 17 years) were recruited. Seventeen teenagers had moved at least once from below and/or above MHBP clinical cut-offs across the cohort study period, 11 had scored above clinical cut-offs at all three waves of data collection, and three had always scored below clinical cut-offs. They had been diagnosed with autism at between 20 months and 8 years (median 42 months). Ten also had a learning disability (IQ < 70). Around half had attended mainstream education only, and just five had attended special education only. Almost all (*n* = 30) were still in full-time education (school or university). Most (*n* = 27) had at least one sibling and seven had at least one other sibling with an autism diagnosis. Over half of mothers (17/30) identified themselves as White British, a third (10/30) as Black British and the remainder described other ethnicities. Over two-thirds were married/co-habiting (24/30). The majority of mothers were working part-time (*n* = 12) or not working (*n* = 11). No further information on socio-economic status was collected. None of the parents identified themselves as autistic.

### Parents’ accounts of their child’s mental health and behaviour problems

Parents’ accounts revealed four broad alternative trajectories of their child’s MHBPs. These did not always align with the cohort data used for sampling:

- MHBPs not reported;
- MHBPs fluctuating in severity across childhood and adolescence;
- MHBPs consistently present;
- MHBPs emerging in the teenage years.

Across these trajectories, parents reported changes in the way these difficulties presented, with a greater diversity, and severity, of MHBPs with age.

Thus, parents’ accounts of early and middle childhood were dominated by descriptions of difficult behaviours (e.g. ‘temper tantrums’, ‘meltdowns’). Reference to emotional difficulties was unusual and when mentioned, typically labelled as ‘anxiety’ or a similar term. Descriptions of late childhood and adolescence, however, saw a shift in parents’ accounts, with increasing reference to concerns about emotional well-being, and greater reference to ‘depression’ and obsessional behaviours. Meltdowns, if present, were more likely to be described as involving harm to others or damage to property. Self-harming, previously presented as part of ‘meltdowns’, was specified separately and was of a more serious nature (i.e. cutting, stabbing). Parents also described increasing concerns around social withdrawal (e.g. reluctance or refusal to leave the bedroom or home) which, if present when the child was younger, had not been regarded as a significant problem. In addition, a number of new MHBPs featured in parents’ accounts. Most common were suicidal thoughts or attempts; eating disorders; video game addiction (i.e. refusal to stop to eat, sleep or wash); and controlling behaviour within the home (e.g. insistence that lights are always switched off, all windows are kept open). Less common were accounts of stealing; running away from home; and viewing and/or posting violent or sexual images on social media. In a few cases, this had resulted in police involvement.

Accompanying these changes were, for parents, increasing levels of concern for, and a pre-occupation with, their child’s welfare and future outcomes.

### Parents’ beliefs about the factors influencing their child’s mental health and behaviour problems

Parents identified a wide range of factors which they believed had caused or influenced improvements or deteriorations in MHBPs, or a maintenance of the status quo. They typically identified more than one factor as influencing a particular MHBP ‘episode’ and articulated the complex, transactional nature of the relationships between factors.

#### Child-centred factors

Autistic traits, verbal communication and intelligence were consistently identified by parents as influencing whether MHBPs were present and the extent to which they could be ameliorated.

##### Autistic traits

Autistic traits were regarded as underlying many of their child’s MHBPs. Similarly, the minority of parents reporting their child had never experienced MHBPs attributed this largely to the fact that their child had mild autistic traits or was only ‘partially’ autistic. ‘Meltdowns’ during early and middle childhood were typically attributed to rigidity around routines and being prevented from engaging in special interests. Some parents also flagged sensory sensitivities, particularly certain noises and noise levels, as triggering anxiety and distress.

I mean we went through the stage of ear defenders, ear plugs, all sorts of things, because even just trying to get from home to childcare he’d be distracted or upset by noises in the street. So it, it was quite traumatic and he’d be distressed for a lot of the time. (ID490)

Social communication differences were regarded as far more salient to MHBPs during adolescence. Difficulties forming and sustaining friendships and, sometimes, experiences of bullying were regarded by many parents to be the primary cause of their child’s low mood and ‘depression’ in the teenage years.

##### Verbal communication

The majority of children had limited or no verbal communication before diagnosis. However, for most, targeted interventions in the post-diagnosis period had addressed this. Having an effective way of communicating with their child was consistently identified as critical to preventing and addressing MHBPs. Similarly, parents with children who did not communicate verbally believed this contributed significantly to angry and aggressive behaviours.

If he’s not able to communicate something, then he would throw tantrums, he will jump and cry and throw things and kick the door. (ID189)

##### Intelligence

Specific to the teenage years, parents who considered their child to be of above average intelligence believed this could worsen MHBPs because of their reasoning abilities. Thus, some parents described their teenager’s skills at rationalising their behaviours to others, or not wanting or valuing the support of others perceived as less intelligent than themselves.

They are too intelligent for you, too intelligent to argue with. (ID277)

In addition, parents believed greater intelligence enabled their child to find ways to engage in damaging or risky behaviour without their parents’ knowledge.

#### Socio-environmental factors

All parents believed multiple aspects of the child’s social environment played a critical role in mental health outcomes. The parents themselves and the school/college environment dominated parents’ descriptions of causal influences of, and the factors moderating, their child’s mental health. Also relevant to some were relationships with non-autistic siblings, school peers, access to social networks beyond school, and the involvement of mental health services. We summarise findings for each in turn.

##### Parents’ capacity and skills in managing mental health and behaviour problems

It was clear from parents’ accounts that many had invested a great deal of time and effort in supporting and nurturing their child. They provided detailed descriptions of, during the post-diagnostic period, learning how to protect against and manage MHBPs through input from autism specialist services, their own information seeking and trial and error. These accounts revealed the highly individual nature of parenting strategies, with parents using a wide range of both generic parenting strategies and those responding to behaviours attributed to autistic trait(s). Here most opted to accommodate autistic traits, but some believed exposure to challenging environments (e.g. supermarket, after school clubs) and *not* adhering to daily routines helped their child to overcome their difficulties.

It was notable that, while almost all parents reported feeling they had become competent in managing their child’s MHBPs in the years following diagnosis, the emergence of new MHBPs in adolescence, or a worsening of existing MHBPs, stripped away that sense of competence and left parents feeling helpless.

I genuinely don’t know what I should be saying, I don’t know what I should encourage, what I shouldn’t encourage. I mean with the self-harming in particular, I Googled it and I read about it and all that kind of thing, but ultimately I, I was, and still am, very worried about doing the wrong thing and making things worse. And so, no, I think actually what I feel is monumentally out of my depth in, in terms of this, yeah. (ID743)

In addition, parents stressed that their capacity (i.e. time, energy) and skills to manage MHBPs were influenced by their own personal circumstances (e.g. physical or mental health, other caring responsibilities, presence of a partner, financial resources) and access to advice and support. With regard to the latter, a common theme was the absence of statutory autism-specialist advice and support once the immediate post-diagnostic period had elapsed. A picture emerged of parents replacing this with self-directed research, commissioning services themselves, and serendipitous contacts with other parents of autistic children.

It’s sad that a lot of what parents do depends on picking up ideas from bumping into other parents in the street. (ID162)

A few parents also acknowledged they had done things which, on reflection, may have exacerbated, or failed to address, their child’s MHBPs (e.g. expressing frustration, avoiding situations, allowing teenagers too much autonomy online and in the real world).

##### Relationships with non-autistic siblings

Generally, parents believed the presence of non-autistic siblings supported the development of the autistic child’s social and communication skills which, in turn supported mental health. Positive sibling relationships could also be an important additional source of emotional support. Indeed, some believed their child confided with their sibling in a way they did not with them. There were also stories of siblings supporting engagement in wider social activities, such as encouraging the autistic child to also join clubs and other organised activities, or going out socially together.

Thankfully he’s always had his brother, and he [brother] is very much an in your face little chap, he always has been, and he never kind of let [autistic child] withdraw. (ID430)

However, not all sibling relationships were positive, with some siblings unwilling to accommodate the autistic’s child behaviour, which served to heighten tension and conflict within the home, in turn worsening the autistic child’s behaviour.

##### Peer relationships

Descriptions of significant positive friendships were relatively unusual. Instead, problems with peer relationships at school were frequently identified as negatively affecting their child, sometimes quite significantly. For some, this included bullying which, particularly when serious (e.g. sustained verbal abuse, physical attacks), had contributed to increased levels of anxiety, panic attacks, and/or eating disorders.

One exception to negative or ambivalent experiences of relationships with school peers was when a child attended a school with a specialist curriculum (e.g. maths, art) that aligned with their interests. In these situations, parents described their child finding ‘like-minded’ peers who were accepting of them. They believed this had reduced anxiety about school attendance.

I think the kids there are just a lot more accepting because they’re all fairly creative and with that it brings, you know, very different characters, and it’s just, well considerably more relaxed, which has worked well for him. (ID430)

##### Social networks beyond immediate family and school

Extended family and family friends, and community groups, were sometimes identified as having played an important role in a child’s mental health. Some parents deliberately nurtured relationships with members of the wider family to extend their child’s sources of emotional support. Community-based clubs and activities (e.g. theatre groups, scouts, dance classes) were regarded as serving to protect mental health by allowing the development of friendship groups outside of school, boosting self-esteem and offering the opportunity to take part in meaningful activities. Befriender services were flagged by a few parents as having a pivotal impact on their child’s mental health and highly valued. Parents also reported that loss of access to such services and activities had resulted in a deterioration, sometimes severe, in their child’s mental health.

I’m sure that if this man [befriender] was still coming he wouldn’t spend all his holidays and every, all the time, on his computer. (ID277)

##### The school environment

Parents’ accounts often dwelt extensively on the different ways school affected their child’s mental health. They believed that, in principle, the learning opportunities and routine and structured nature of the school day meant attending school should be supportive of positive mental health. However, their actual experiences were often very different or, at best, mixed: it was regarded as a critical environment to get right.

Parents identified multiple ways in which a school environment increased the risk for, or mitigated against, MHBPs. These were

- degree of autism specialism;
- opportunities for pupils to engage in subjects and activities that interest them;
- policies and practices around managing behaviour problems;
- nature of the sensory environment;
- consistency of staff;
- academic expectations;
- support provided to parents.

Some parents described changing their child’s school because of the significant negative impacts of the school environment on their child’s MHBPs. Most reported dramatic improvements following such a move.

I got him in and he never looked back. It was the best thing. I couldn’t, couldn’t believe it, he went from strength to strength when he got in there. (ID186)

However, there were exceptions, usually when a young person without learning difficulties moved from a mainstream to a special school setting. Importantly, parents with experiences of both mainstream and special schools identified advantages and risks in both settings.

Within our sample, *autism-specialist* mainstream and special schools were more likely to make use of autism-specific accommodations (e.g. visual timetables, social stories) and parents believed these helped to minimise anxiety and agitation. The specialist nature of these schools also meant there were a number of autistic children or young people. Parents believed that an important consequence of this was, within the school, a greater acceptance, and even celebration, of neurodiversity. Parents with experience of such an ethos, or atmosphere, regarded this as being highly beneficial.

Parents spoke about schools that offered their child *opportunities to engage more fully in subjects and activities they were interested in* as being good for their mental health and wellbeing. This was described by parents of children attending secondary schools/colleges specialist in a particular academic subject, or special schools that made efforts to ensure their timetable included activities they enjoyed.

For the first time in his life it was like he took off a really uncomfortable pair of shoes and relaxed and then, at the age of sixteen, he just became quite a nice person and a lot of the anger just dissipated overnight. (ID409)

Special schools were typically regarded as more skilled at *managing behaviour problems*, and parents believed the rewards-based approach usually employed in these settings to be effective. However, some parents attributed the emergence of behaviour problems to classmates’ behavioural difficulties and the way staff responded to them.

[name of son] told me that these children would behave badly and the teachers won’t do anything to like sanction or reprimand them or anything, just take them round for a walk to calm them down. [name of son] won’t do that in school, but he will come home and practise that on me and that is not good enough. (ID6)

In contrast, mainstream schools were presented as sometimes reacting too quickly to small misdemeanours and being overly punitive in their response (e.g. teaching the child separately on a long-term basis, excluding them from specific extra-curricular activities, and/or placing them ‘on report’).

I felt this is just excluding, you’re being cruel, you’re not even trying to work with him, or even understand him. (ID186)

Where this had happened, parents consistently reported deteriorations in their child’s mood and behaviour.

However, special schools were regarded as being a more challenging *sensory environment*. In particular, the large numbers of pupils with behavioural problems meant that they were regarded as noisier and less predictable environments, thus carrying the risk of increasing anxiety. Indeed, in some cases, this had led to school refusal.

*Consistency of staff* was another feature of the school environment regarded as an influence on mental well-being, increasing agitation and anxiety and, in some instances, leading to school refusal. As well as the challenges to the child of coping with unfamiliar faces, high rates of staff turnover led to inconsistencies in knowledge and practice around autism-specific accommodations and behaviour management strategies.

Parents were appreciative of schools which supported their child to make progress academically. However, during the teenage years, *academic expectations* were frequently identified by parents as affecting their child’s mental health. This was the case regardless of the type of school, but particularly acutely experienced in mainstream schools in GCSE and A’level years.

She had to write this timed essay and she couldn’t do it, she was sitting under the table, rocking, crying. I couldn’t get her out of the house. She was completely and utterly overwhelmed and depressed. (ID326)

A final, and important, influence of schools on MHBPs identified in by parents’ was the role schools played in *supporting parents*. Indeed, many described working in partnership with teachers to help prevent and manage their child’s mental health and behaviour problems, both at school and at home. Key features of effective partnerships were respect, communication and joint decision-making.

We kinda just gelled and worked together . . .. they kind of wanted our input. They’d send home a little report and update you. (ID6)

##### Access to mental health services

A lack of access to statutory mental health services was a dominant theme in parents’ accounts. Some recoursed to private providers – either for parenting/behaviour management advice or direct treatment/therapy for the child – though the cost meant this was not an option for some.

We have tried time and time again through the GP to get her referred for counselling or some kinda programme to help, particularly with the eating disorder. And, yeah, every time we’ve drawn a blank. We did look at doing it privately but, like I say, they want her as an inpatient and it’s about six grand a week so that’s probably just not feasible. (ID733)

Among those who had accessed statutory mental health services, experiences were mixed. Some children had never attended appointments or dropped out rapidly, either because they did not accept they needed mental health support, or the requirement to attend appointments at clinics proved a significant barrier due to a reluctance to leave their home or struggles with public transport.

How can they help a person like [name of son] when he is not sort of admitting to his, in denial of his condition? Not going to appointments, not doing anything. (ID277)

The effectiveness of interventions offered was variable.

#### Developmental changes

The process of recalling experiences of MHBPs across their child’s life facilitated parents’ reflections on the impacts of developmental changes. These were believed to have either caused new MHBPs or affected the presence or severity of existing MHBPs.

##### Physical and sexual development

Some parents believed their child had become more volatile (e.g. more frequent and severe ‘angry outbursts’) during puberty. These were attributed to hormonal changes, as well as the child’s struggles with physical changes and the development of sexual feelings.

I always say it was as if the change in hormones woke him up. It’s like he just seemed to be oblivious to everything, and then all of a sudden he just became aware and he just couldn’t cope. (ID62)

In some cases, increasing size affected how parents responded to difficult behaviours with physical strategies no longer possible. Furthermore, a minority reported becoming fearful of their child’s aggressive behaviours and avoided confrontations (indeed, some had been injured by their child on one or more occasions). In some instances, this had led to the child exerting a high degree of control over what happened in the home (e.g. noise and light levels) and parents had ceased trying to address concerning behaviours (e.g. a perceived addiction to video gaming).

##### Psychosocial development: identity development and the autism diagnosis

Many parents of children without learning disabilities described adolescence as a time when they became more aware of their autism diagnosis. There were multiple reasons for this. Some parents had withheld the diagnosis until the child was a teenager. Others, particularly those in mainstream education, believed that differences between themselves and neurotypical peers became increasingly apparent. This heightened consciousness led some young people to ‘research’ autism; in all instances, this had not proved helpful.

Crucially, parents reported that their child’s perception of their autism impacted their mental health. In most cases, their child’s feelings about being autistic were, or became, entirely negative and they struggled to find ways of positively incorporating it into their sense of identity. Feelings of anger, embarrassment and anxiety linked to wanting to hide the diagnosis from others were frequently described.

She hates it, she’s ashamed of it, she’s embarrassed about it, she has no self-esteem because of it. (ID165)

In some cases, parents attributed self-harming and suicidal ideation to their child’s struggles with the autism diagnosis and its implications. Furthermore, some had found that a lack of acceptance made it difficult for others (e.g. family, professionals) to support the young person.

I would say that once he went into the senior school it changed . . . He didn’t want any kind of association with that [being autistic],. . . he was very much sort of I don’t want, I don’t want assistants around me, I don’t want anybody to mention it. That was difficult, that was really difficult, because he just kept pushing help away. (ID430)

Occasionally, young people were said to have come to terms with the diagnosis or to view being autism as positive, either through the parent’s efforts to promote it as such or due to interventions by school counsellors. Parents felt this had been beneficial as it had enabled their child to talk to friends and family about what being autistic was like for them, which in turn led to greater acceptance of the young person’s autistic behaviours and reduced conflict.

##### Psychosocial development: developments in social skills

Occasionally parents reported their child had become more comfortable and confident in social situations, particularly with peers, as they grew older. Indeed, some parents reported newfound interest in spending time with friends during the teenage years.

He suddenly sort of socially has come out of his shell more. He does go out with people from school to, occasionally to sleep over or the cinema, which he’s never done, ever, before. (ID427)

Parents attributed this to developments in the child’s social skills, caused both by a greater ability to express emotions and empathise with others and cumulative social experiences. They believed increased and easier contacts with peers had positively impacted depressive symptoms and feelings of loneliness.

##### Psychosocial development: developments in self-management strategies

By the time they reached teenage years, many parents reported their child had developed effective strategies to manage sensory sensitivities, dislike of change and uncertainty, and heightened stress. As a result, by this life stage, some autistic traits were less likely to be regarded as influencing MHBPs, particularly anxiety.

He’s learned how to manage things. Like, for example, he’ll be watching something on YouTube and he knows that there’s a bit coming up that he doesn’t like but he still has to watch it; so he’ll either turn the volume down or he’ll cover his ears and vocalise. (ID62)

#### Life events: bereavement(s)/separation(s)

Experiences of bereavement (e.g. death of family member or friends) and separation, both temporary (e.g. family member hospitalised) and more permanent (e.g. parental separation, siblings leaving family home), were regarded by parents as potential causes of a deterioration in mental health. While acknowledging such events may impact any young person, parents felt they were more significant for their children for two reasons. First, their social networks were more limited and therefore loss or separation from key sources of social and emotional support more keenly felt. Second, loss or separation resulted in changes and uncertainties at home, something which most parents said their child found challenging.

Fifty percent of his difficulties are caused by not knowing what is going to happen. So [his] mum being away [for medical treatment] and then taking longer to recover than expected was a problem. He doesn’t fully understand why she can’t do the things she usually does. (ID25)

## Discussion

Much existing research investigating autistic children’s mental health positions clinicians and academics as the (sole) experts. This study positioned parents as experts, seeking to elicit their accounts and understandings of their child’s mental health and their ‘theories’ on the factors affecting it. Aside from studies on transition, research with parents of autistic teenagers is extremely limited. This is a critical omission given autistic teenagers are typically invisible to clinicians unless there are significant and acute concerns about their mental health. This article therefore brings a new perspective and, in doing so, has implications for both research and practice.

The range of factors which parents believed had impacted their child’s mental health are described, as well as their theories on whether such factors had a direct and/or indirect effect, and the complex and sometimes transactional nature of the relationships between factors.

In line with existing evidence (Carter Leno et al., 2021; Carter Leno et al., 2018; McCauley et al., 2020; Milosavljevic et al., 2016; Woodman et al., 2016), parents believed a number of child-centred factors (i.e. autism traits, intelligence, verbal communication skills) played a fundamental role in their child’s mental health. Developmental factors also featured strongly in parents’ accounts, identified as either a threat to, or serving to ameliorate or protect against, mental health difficulties. To date, few studies have included measures of physical, sexual or psychosocial development.

A third domain of influence on mental health outcomes identified by parents were socio-environmental factors. These were located within the family (parents, siblings), social networks beyond immediate family and school, school peers and features of the school environment itself. In terms of parent-centred factors, it was notable that most parents opted to accommodate their child’s autistic traits, but a few took the opposite approach, exposing their child to challenging situations to help them overcome difficulties. In both cases, there were some parents who expressed uncertainty, and occasionally regret, at the approach adopted. At present, research evidence on the impact of accommodation on MHBPs is minimal and this is an issue that clearly warrants attention (O’Nions et al., 2020).

Aside from parent-centred factors, parents believed the school environment exerted the greatest influence on their child’s mental well-being and, aligning with previous studies (Falkmer et al., 2015; Starr & Foy, 2010), a number of different features were identified as relevant. Importantly, the school environment not only directly impacted their child’s mental health, but it also supported or hindered their efforts, as parents, to nurture and care for their child. While observational studies have considered the impact of socio-environmental factors on mental health outcomes, the focus is often on (some) parent-centred factors (Dieleman et al., 2017; Emerson et al., 2014; O’Nions et al., 2020; Woodman et al., 2016; Yorke et al., 2018). The absence of research investigating school environment is striking. Indeed, where studies have included an indicator of school environment, this is typically limited to type of school (i.e. mainstream vs special) (Reed et al., 2012; Simonoff et al., 2013; Simonoff et al., 2020; Stringer et al., 2020).

Finally, many parents’ accounts included descriptions of life events involving bereavement and separation which had adversely affected their autistic child. Parents believed that, compared to neurotypical children, autistic children were at greater risk because the consequence of such life events is either change and uncertainty or disruption to (often more limited) social networks. Such beliefs certainly align with other work reporting autistic adults to perceive such life events as more stressful than non-autistic individuals (Bishop-Fitzpatrick et al., 2017; Rumball et al., 2020; Taylor & Gotham, 2016).

In terms of implications, for research, the findings highlight the value and importance of bringing parents’ expertise to understanding the factors and processes affecting autistic children’s mental well-being. Their accounts support previous calls for research to be grounded in a transactional model of development (Binns & Oram Cardy, 2019; Mills et al., 2022; Stallworthy & Masten, 2022). Within this, parents identified a wide range of child-centred, developmental, socio-environmental and life event factors, many of which have received little or no research attention. Parents’ views on their relative importance should inform what is prioritised by future studies and we note the dominance of the school environment in many parents’ accounts. To develop work in this area will require the specification and development of appropriate indicators.

There are also implications for policy and practice. Two strong themes in parents’ accounts were self-directed information-seeking (including, for some, ‘purchasing’ of interventions) prompted by a lack of access to autism specialist support and, in the early teenage years, a loss of perceived competence in managing emotional and behavioural difficulties, and uncertainty around how to best support their child’s mental well-being. These findings support recent calls for autism services to be modelled on approaches adopted by other long-term conditions (Green, 2019), including equipping and upskilling parents in the post-diagnosis period and ensuring easy access to stepped-up care.

The findings also indicate that, for some, the autism diagnosis can challenge identity development, impacting (sometimes quite significantly) mental well-being. In line with previous studies (Cresswell & Cage, 2019; Mesa & Hamilton, 2022; Mogensen & Mason, 2015; Riccio et al., 2020), our findings support calls for advice and support for parents on explaining/disclosing an autism diagnosis; psychoeducational interventions for autistic teenagers themselves; and guidance for schools on nurturing positive perspectives on neurodiversity.

In addition, aspects of the school environment (i.e. school ethos, policy and practice) were consistently identified as having the potential to significantly impact mental health outcomes, either positively or negatively. The ambition of the UK government policy (‘The National Strategy for Autistic Children, Young People and Adults: 2021 To 2026’) is for more autistic children to have positive school experiences and it is targeting improvements in understanding of autism among education professionals, anti-bullying programmes and the involvement of young people and parents in re-designing special needs systems and provision. It is too early to comment on the impact of these initiatives, but a policy intervention such as this does have the potential to ensure widespread improvements.

Finally, parents described barriers to young people accessing mental health services and needing greater support to engage with such services. This accords with calls (Brice et al., 2021) to remove or minimise the barriers to accessing health services. The option of online mental health care brought about by COVID may go some way to addressing this issue. However, further research is needed to allow evidence-informed decision-making about how care is provided.

### Study limitations

The majority of participants were mothers. Fathers may have different perspectives which would help us further understand the factors and processes which impact mental health outcomes. The study sought parents’ retrospective accounts from early childhood onwards. Research capturing ‘in the moment’ beliefs and theorising across the span of childhood and teenage years would be a useful development to this research. Equally important is research with autistic children and teenagers themselves. Finally, the sample was recruited from families living in London when their child was first diagnosed. It is unlikely these represent the range of types of school environment and autism-specialist support services present in the United Kingdom.

## Supplemental Material

sj-docx-1-aut-10.1177_13623613231158959 – Supplemental material for Factors influencing the mental health of autistic children and teenagers: Parents’ observations and experiences Click here for additional data file. Supplemental material, sj-docx-1-aut-10.1177_13623613231158959 for Factors influencing the mental health of autistic children and teenagers: Parents’ observations and experiences by Suzanne Mukherjee and Bryony Beresford in Autism

sj-docx-2-aut-10.1177_13623613231158959 – Supplemental material for Factors influencing the mental health of autistic children and teenagers: Parents’ observations and experiences Click here for additional data file. Supplemental material, sj-docx-2-aut-10.1177_13623613231158959 for Factors influencing the mental health of autistic children and teenagers: Parents’ observations and experiences by Suzanne Mukherjee and Bryony Beresford in Autism

sj-docx-3-aut-10.1177_13623613231158959 – Supplemental material for Factors influencing the mental health of autistic children and teenagers: Parents’ observations and experiences Click here for additional data file. Supplemental material, sj-docx-3-aut-10.1177_13623613231158959 for Factors influencing the mental health of autistic children and teenagers: Parents’ observations and experiences by Suzanne Mukherjee and Bryony Beresford in Autism

sj-docx-4-aut-10.1177_13623613231158959 – Supplemental material for Factors influencing the mental health of autistic children and teenagers: Parents’ observations and experiences Click here for additional data file. Supplemental material, sj-docx-4-aut-10.1177_13623613231158959 for Factors influencing the mental health of autistic children and teenagers: Parents’ observations and experiences by Suzanne Mukherjee and Bryony Beresford in Autism
