# Supplementary material for: Factors influencing the mental health of autistic children and teenagers: Parents’ observations and experiences
Source: Autism. 2023 Mar 15;27(8):2324–36. doi: 10.1177/13623613231158959 (PMC10576903; doi:10.1177/13623613231158959)
Supplement: sj-docx-1-aut-10.1177_13623613231158959 – Supplemental material for Factors influencing the mental health of autistic children and teenagers: Parents’ observations and experiences [file sj-docx-1-aut-10.1177_13623613231158959.docx]

**Supplementary file 1:**

| **Mental Health Trajectory** | **Gender** | **IQ<70** | **IQ>70** | **Total Completed** |
| --- | --- | --- | --- | --- |
| **Early improver**  (T1 high MHBP, T2 low MHBP, T3 low MHBPS) | ***Males*** | 0/4 | 2/9 | 3/19 |
|  | ***Females*** | 0/3 | 1/3 |  |
| **Late improver**  (T1 high MHBPS, T2 high MHBPs, T3 low MHBPS) | ***Males*** | 1/3 | 3/12 | 4/20 |
|  | ***Females*** | 0/5 | 0/0 |  |
| **Early decliner**  (T1 low MHBPS, T2 high MHBPS, T3 high MHBPS) | ***Males*** | 0/1 | 2/4 | 2/5 |
|  | ***Females*** | 0/0 | 0/0 |  |
| **Late decliner**  (T1 low MHBPS, TS low MHBPS, T3 high MHBPS) | ***Males*** | 0/0 | 1/3 | 1/4 |
|  | ***Females*** | 0/0 | 0/1 |  |
| **Positive path with T2 switch**  (T1 low MHBPS, T3 high MHBPS, T3 low MHBPS) | ***Males*** | 1/3 | 0/1 | 4/8 |
|  | ***Females*** | 0/1 | 3/3 |  |
| **Negative path with T2 switch**  (T1 high MHBPS, T3 low MHBPS, T3 high MHBPS) | ***Males*** | 0/5 | 2/10 | 3/17 |
|  | ***Females*** | 0/0 | 1/2 |  |
| **Consistently report low MHBPS** | ***Males*** | 0/3 | 1/6 | 3/11 |
|  | ***Females*** | 0/0 | 2/2 |  |
| **Consistently report high MHBPS** | ***Males*** | 7/26 | 1/7 | 11/42 |
|  | ***Females*** | 1/4 | 2/5 |  |
| **TOTAL** |  | **10/58** | **21/68** | **31/126 complete** |
| *NB. 35/126 responded positively to invitation to take part in the research = 27.8 % response rate* | | | | |

**Sampling matrix displaying interviews completed out of total sampling pool**
