# Supplementary material for: Factors influencing the mental health of autistic children and teenagers: Parents’ observations and experiences
Source: Autism. 2023 Mar 15;27(8):2324–36. doi: 10.1177/13623613231158959 (PMC10576903; doi:10.1177/13623613231158959)
Supplement: sj-docx-2-aut-10.1177_13623613231158959 – Supplemental material for Factors influencing the mental health of autistic children and teenagers: Parents’ observations and experiences [file sj-docx-2-aut-10.1177_13623613231158959.docx]

**Supplementary file 2:**

**Extract from interview topic guide**

**Part 2: Main Narration – The parent’s story of the changes in their child’s moods, behaviour, and mental health during their childhood and teenage years**

**Introduction**

- I’d now like to ask you a little bit about [name of child] moods and behaviours have changed over their childhood and teenage, and why you think this might be.
- I am interested in both the ups and downs in their moods and behaviours – in other words both times when things have been going well and times when there have been difficulties.
- We will start with their early childhood, when [NAME OF CHIILD] was first diagnosed and you became involved with the QUEST project.
- I am going to try not to interrupt too much – this is your time to tell me your story.

**Notes to interviewer**

- *Researcher to restrict self to active listening with minimal encouragers (i.e. hmm, yes, I see etc.).*
- *If the participant gets stuck on an issue/event or time period, encourage them to move on with prompts ( i.e. What happened then? When did that change? What happened next? etc.)*
- *Summarise parent’s story, including any reasons for changes, and seek clarification where necessary.*
