# Supplementary material for: Factors influencing the mental health of autistic children and teenagers: Parents’ observations and experiences
Source: Autism. 2023 Mar 15;27(8):2324–36. doi: 10.1177/13623613231158959 (PMC10576903; doi:10.1177/13623613231158959)
Supplement: sj-docx-3-aut-10.1177_13623613231158959 – Supplemental material for Factors influencing the mental health of autistic children and teenagers: Parents’ observations and experiences [file sj-docx-3-aut-10.1177_13623613231158959.docx]

**Supplementary file 3:**

**The data analysis process**

**Step 1: Data immersion and identification of emergent themes**

- Within 24 hours of completing an interview, Researcher 1 (R1) wrote a summary of the interview based on detailed notes made during and immediately after the interview (Note-taking was possible because all but one interview was conducted via telephone). This included field notes, plus a ‘pen portrait’ (Holloway and Jefferson, 2000) summarising the content of the interview and the main themes emerging.
- After completing this task, R1 met with Researcher 2 (R2) to discuss the interview. This served both as a debrief and as a means of ensuring that both researchers were fully familiar with the interview data and themes emerging.
- As transcripts were received back from the transcription agency, they were checked by R1 for accuracy and any necessary changes/additions were also made to the interview summaries.

**Step 2: Development of analytic framework**

- Around halfway through fieldwork, interview summaries were scrutinised by R1 who then produced an initial draft of the analytical, or coding, framework. This included codes relevant to *a priori* and emergent themes. This process was informed by the transactional model of development.
- R1 and R2 then met a number of times to review and refine the framework during the second phase of fieldwork, including the labels assigned to codes and the grouping of codes under higher order themes/concepts.

**Step 3: Testing of coding framework and initial data extraction**

- Once fieldwork was complete, the coding framework was finalised and entered into data extraction template (created in EXCEL), with each code a column and each row representing a participant. The left-hand column used to enter relevant socio-demographic and autism-related information on the research participant and their child. the most right-hand column was used for researcher observations.
- The coding framework and data extraction template was then tested on a sub-sample of interview transcripts (n=10).
- First, R1 read through each transcript line by line and applied codes to the text.
- Coded data was then extracted into the EXCEL spreadsheets in the form of summary statements and verbatim quotes.
- If data was identified in the transcript that could not be coded to the existing framework, this was extracted into a new column and tentative code label created.
- After completing coding and data extraction on 10 transcripts, R1 and R2 met to review the data extraction charts and identify ways in which the coding framework needed to be revised. Aside from adding new codes, no significant changes were made to the coding framework.

**Step 4: Data extraction, reduction and display of total data set**

- R1 completed then data extraction on the remaining interview transcripts.
- Where new codes were added, checks were made of coded and extracted transcripts to see if there was any data relevant to new codes. This was then coded and extracted into the study participant’s data extraction.
- R2 reviewed the final data extraction for sense, comprehensiveness and consistency.

**Step 5: Analytical writing and data display**

1. R1 used the data contained within the extraction template to create extended analytical notes describing the data contained in each code. As part of this process, the data for each participant was examined to identify any patterns or changes over time (i.e., were some experiences specific to early childhood, adolescence etc.). In addition, comparisons were made *between* study participants to identify any differences according to participant characteristics (i.e. young people with and without LD, males and females).
2. At iterative process involving review of analytical notes and discussion within the team (R1, R2) refined these analytical notes. Diagrams were also created to display observed (inter)connections between factors identified by parents as impacting their child’s mental health (e.g., school environment and parenting strategies), observed changes in the relevance of factors over time, and more distal factors identified as playing a role (e.g. parental health and parenting strategies). Project outputs were written from these sources.
