# Supplementary material for: Factors influencing the mental health of autistic children and teenagers: Parents’ observations and experiences
Source: Autism. 2023 Mar 15;27(8):2324–36. doi: 10.1177/13623613231158959 (PMC10576903; doi:10.1177/13623613231158959)
Supplement: sj-docx-4-aut-10.1177_13623613231158959 – Supplemental material for Factors influencing the mental health of autistic children and teenagers: Parents’ observations and experiences [file sj-docx-4-aut-10.1177_13623613231158959.docx]

**Supplementary file 4:**

**Nodal framework for data extraction and coding**

**The child**

- Early autism markers that led to diagnostic assessment
- How autism affects the child

**The parent**

- The skills and experience the parent brings to the role
- The development of knowledge and understanding about autism

**The context in which parents are caring for a child with autism**

- Other caring responsibilities
  - Other children who need attention and support
  - Another child who has significant care needs due to illness/disability
  - Other family member with care needs (e.g. partner, parent etc.)
- Practical help/support available to parents in their parenting role
  - Partner
  - Extended family and friends
  - Statutory services (e.g. respite, mentors, carers etc.)
- Financial resources
- Stressful life events
  - Serious illness within immediate family
  - Bereavement
  - Separation

**The parent’s experience of child’s mental health and behaviour problems and impact on family**

- The child’s mental health and behaviour
  - The nature of the child’s mental health and behaviour problems (MHBPs) and its trajectory over the years
  - The time periods parents have found most challenging
  - The MHBPs that most concerns parents
- Impact of child’s MHBPs on parent
- Impact of child’s MHBPs on wider family
- Parent’s outlook on the child’s future
- Support needs identified by parent
  - to support child
  - to support parent & wider family
- Support refused by parents/ child

**Parent’s views on factors that influenced the child’s mental health and behavioural trajectory**

*N.B. For each factor, will code separately things that have: (1) reduced or stablished MHBPs (2) things that have not helped/made no difference (including services that have been difficult to access); (3) that have worsened MHBPs.*

- **The child**
  - Autistic traits
  - Verbal ability
  - Intelligence/mental age
  - Developmental stage/puberty/hormones
  - Child’s awareness of & response to autism diagnosis
  - Child’s own self-management strategies
  - Private therapists
- **The home environment**
  - Parent’s reaction to autism diagnosis
  - Parent’s strategies for managing MHBPs:
    - Accommodation
    - Positive reinforcement
    - Minimising uncertainty
    - Relaxation techniques
    - Encouraging/supporting activities & interests
    - Efforts to block activities (e.g. gaming)
  - The role of partners in managing MHBPs
  - Sibling relationships
  - Change and loss (e.g. illness, bereavement, separation etc.)
  - The physical environment
- **The school environment**
  - The demands and expectations the school places on children
  - School accommodation of the autistic child
  - School strategies for managing MHBPs
  - School communication and relationship with parents
  - Peer relationships
  - The school as a source of structure & activity
  - Change and loss (e.g. bereavement, change of teacher etc.)
  - The physical environment
- **Other statutory services**
  - Statutory mental health services (e.g. Child and adolescent mental health services, Adult psychiatric services etc.)
  - Speech and language therapists
  - Mentors
  - Private therapists
